# Bibliographical Notices

**Published:** 1844-04-20

**Authors:** 


### BIBLIOGRAPHICAL NOTICES.

*The Cyclopædia of Practical Medicine.* Edited by John Forbes, M.D., F. R. S., Alexander Tweedie, M. D., F. R. S., and John Conolly, M. D. *Revised, with Additions.* By Robley Dunglison, M. D. Part I. Philadelphia: Lea and Blanchard. 1844. Royal Octavo.

This work consists of original articles on all the important subjects of medical science, contributed by many of the most eminent British Physicians, including Professors and distinguished Teachers in London, Edinburgh, Dublin, and Glasgow; gentlemen of great learning and experience in the profession, and known as successful cultivators of the several departments on which they have written. No work of the kind has issued from the press with better promise, or has more fully accomplished the objects proposed.

Part 1st, now published, contains twenty-three articles, by nearly as many different authors. These, although written with a brevity consistent with the design of the work, display much learning, judgment, and discrimination. The present number may be considered as a fair sample of the whole, and in that view cannot fail to be acceptable to those who may desire to possess such a work. It contains 152 pages, printed in double column, with new type, and on good paper.

The publishers announce that one *part* is to be issued every two weeks, until the whole is completed, which will be comprised in twenty-four parts; so that purchasers may expect to be in possession of the entire work in less than a year: a period quite brief enough, it must be admitted, for the accomplishment of such an undertaking. However, they are business men, with ample means; and the Editor, it is well known, is every way qualified for the task he has assumed; so that no apprehension need be felt for the punctual performance of all that is promised.

*Fifteenth Annual Report of the Inspectors of the Eastern State Penitentiary of Pennsylvania. Read in the Senate and House of Representatives, March,* 1844.

It is doubtless known to most of our readers that the system pursued in this prison is that of solitary confinement, the prisoners being completely isolated, except as to the occasional visits of particular officers.

No one, we think, can well dispute the superiority of this system over every other, so far as the suppression of crime and reformation of the convict is concerned; but the probable influence of such a treatment, under the most humane administration, on the health of the prisoners, physical and mental, has been the subject of much anxious thought and eloquent disquisition.

Nothing, it is clear, can solve the problem and quiet the fears of the benevolent, but the result of experiments fully and fairly carried out. On this account, the Annual Reports of the two Penitentiaries of Pennsylvania, are always deeply interesting to the Philanthropist as well as to the Physician and Legislator.

Thus far, experience has certainly sustained the hopes of the advocates of the solitary system, and greatly lessened the apprehensions of many who were once its opponents. The present Report may indeed be considered as almost conclusive, in regard to the points heretofore most in dispute.

It appears that: « The whole number of deaths during the past year was 11. Of these, 5 were white, and 6 coloured. The whole number of prisoners during the year, was 487; of these, 325 were white, and 162 coloured; showing a per centum of mortality of 1.53, as to the white, and 3.66, as to the coloured prisoners."

« The number of deaths among white and coloured was the same."

« There were admitted into the prison since the 1st day of January, 1843, 60 white prisoners, and 23 coloured prisoners, in good health; and 53 white, and 20 coloured prisoners, in imperfect health.

Two white prisoners, of the 53 admitted in imperfect health, were in the last stage of pulmonary consumption, under which they had been labouring for many months."

These facts, proclaimed by the inspectors, certainly exhibit no unusual mortality among the prisoners, and when we consider the habits of such persons prior to their admission, the proportion of deaths appears to us to have been remarkably small.

The means employed by the intelligent physician to promote the health of the prisoners, he has stated as follows: « Strict cleanliness of person and cell, free ventilation, abundance of clean and well dried bedding, regular exercise in the cell yards, moderate diet, a comfortable temperature, clean seasonable clothing and constant occupation,—have been at all times impressed on the officers as the surest means of preserving the health of the objects of their care."

In the opinion of Dr. Edward Hartshorne, physician of the Institution, the « more general use of flannel under-garments has had a remarkably salutary influence, which is fully shown by the diminished frequency and violence of rheumatisms, neuralgic-pains, colds and other disorders of that kind."

"The acute diseases have been," he observes, "with few exceptions, mild, and of short duration. They are similar in character, but appear to be, in general, less frequent and severe than among artisans of analogous occupations in the city. The chronic diseases have been, as heretofore, principally diseases of the digestive apparatus, rheumatic, neuralgic, and venereal affections: together with some tuberculous disease in the shape of scrofulous inflammation and phthisis. The last two are rare, except among the coloured population, and constitute among them a considerable amount of introduced incurable disease."

"Some of the cases of visceral disease are probably also due to vicious practices; but a large proportion of them must be attributed to the previous habits of the patients, who are so often found to have ruined their constitutions long before committed."

Dr. Hartshorne declares that he has "not been able to discover any disease peculiar to this penitentiary. Instances of general debility, resulting from various depressing causes, must of course sometimes occur; but they present an assemblage of symptoms sufficiently often met with in other prisons, and elsewhere in public and private practice."

"The amount of tuberculous disease developed in this institution has, thus far, within my sphere of observation, been much less than is generally imagined. Out of nine cases of scrofula, not more than one or two at furthest could be fairly laid to the account of any cause operating in confinement. Of eight cases of phthisis, only three appeared to have been developed in the cells. Two of these latter occurred in wretched subjects, and were evidently due, in a great degree, to causes existing before conviction. But one recent case has occurred among the whites since January last; the subject of this, entered in bad health and extremely low spirits, and being predisposed to consumption, soon presented well-marked symptoms. Nor has more than one case transpired among the coloured inmates during the same interval."

So far from the circumstances under which the prisoners in the Eastern penitentiary are placed, being unfavourable to health, it would appear that the mortality has been about the average of our city. From data obtained from authentic sources, the mortality among the population of Philadelphia, last year, is stated to have been:

| | | |
|---|---|---|
| - - - | White, | 1.93 |
| | Coloured, | 2.70 |
| Eastern Penitentiary, | White, | 1.57 |
| | Coloured, | 3.66 |

In regard to the "influence of separate confinement on the mind," Dr. H. says that, from what he has seen, he has no reason to believe that it "may excite mental alienation in a criminal previously of sound mind."

"The circumstances under which many persons are committed to the cells, would seem to be sufficiently trying to overthrow a sensitive or feebly constituted mind, without the aid of other sinister influences. Yet the amount of continued mental distress, is much less than many inquirers would expect to find. The majority, unless agitated with the hope of pardon, sooner or later become reconciled to their fate, and support its privations with rational fortitude."

"Instead of stultifying the intellect, as some imagine, I am fully satisfied that separate imprisonment is more apt to produce the opposite effect. The perceptions are evidently rendered more acute by coutinued exertion under the difficulties of restraint. The reflective powers also are increased by their unwonted activity under the exigencies of a seclusion, uninterrupted except by intercourse with intelligent and considerate men.

"The acuteness of perception among criminals is proverbial in all prisons; and the experience of this one long since proved that these faculties are by no means blunted there. Nor are the statements in regard to the subdued temper, and improved habits of reflection among separate convicts, mere idle assertions; as all those who have watched the mental developments in this place must be prepared to admit."

